# Value of low-dose dobutamine stress echocardiography on defining true severe low gradient aortic stenosis in patients with preserved left ventricular ejection fraction

**DOI:** 10.1007/s10554-018-1416-z

**Published:** 2018-07-23

**Authors:** Dan Liu, Kai Hu, Eva Liebner, Frank Weidemann, Sebastian Herrmann, Georg Ertl, Stefan Frantz, Peter Nordbeck

**Affiliations:** 1 Department of Internal Medicine I, University Hospital Würzburg, Würzburg, Germany; 2 Comprehensive Heart Failure Center, Würzburg, Germany; 3 Medizinische Klinik I des Klinikum Vest, Recklinghausen, Germany; 4 Medizinische Klinik und Poliklinik I, Comprehensive Heart Failure Center, University Hospital Würzburg, Oberdürrbacher Str. 6, 97080 Würzburg, Germany

**Keywords:** Aortic stenosis, Stress echocardiography, Mitral annular plane systolic excursion, Systolic pulmonary artery pressure, Aortic valve velocity ratio

## Abstract

Low-dose dobutamine stress echocardiography (DSE) is a valuable tool to distinguish true-severe (TS) from pseudo-severe (PS) low gradient aortic valve stenosis (LGAS) in patients with reduced left ventricular ejection fraction (LVEF). However, only scanty studies reported the clinical utility of DSE in differentiating TS-LGAS patients with preserved LVEF. We investigated the clinical utility of DSE in LGAS patients with preserved LVEF and the echocardiographic determinants suggestive of TS-LGAS. 130 consecutive LGAS patients [indexed aortic valve area (AVA) ≤ 0.6cm^2^/m^2^ and mean trans-aortic pressure gradient (PG_mean_) < 40mmHg] with preserved (≥ 50%, n = 63) and reduced (< 50%, n = 67) LVEF were included. DSE defined TS-LGAS (projected AVA ≤ 1 cm^2^) in 61.2% patients with reduced LVEF and in 68.3% patients with preserved LVEF. Multivariate logistic regression analysis showed that baseline AVA was an independent determinant of TS-LGAS both in LVEF ≥ 50% (OR 0.45, P = 0.004) and LVEF < 50% groups (OR 0.55, P = 0.005). Reduced septal and lateral mitral annular plane systolic excursion (MAPSE, OR 0.72 and 0.75, P = 0.013 and 0.016) and septal TDI-s´ were significantly associated with TS-LGAS in patients with LVEF ≥ 50%. Higher systolic pulmonary artery pressure (SPAP, OR 1.43, P = 0.045) was associated with TS-LGAS in patients with LVEF < 50%. DSE is useful to define TS-LGAS also in patients with preserved LVEF. Lower baseline AVA values are linked with TS-LGAS in both patients with reduced and preserved LVEF. Reduced MAPSE and septal TDI-s´ are suggestive of TS-LGAS in patients with preserved LVEF, while higher SPAP is associated with TS-LGAS in patients with reduced LVEF.

**Electronic supplementary material:**

The online version of this article (10.1007/s10554-018-1416-z) contains supplementary material, which is available to authorized users.

## Introduction

Severe aortic stenosis (AS) is usually defined as an aortic valve area (AVA) < 1.0cm^2^, mean transvalvular gradient (PG_mean_) ≥ 40 mmHg, and a peak flow velocity ≥ 4.0 m/s [1]. Patients with AVA < 1.0cm^2^ and PG_mean_<40 mmHg are usually defined as low-gradient AS (LGAS). Further examinations are usually required to distinguish true-severe (TS) from pseudo-severe (PS) AS, which is clinically essential for therapeutic decision making because patients with TS-LGAS might benefit, whereas PS-LGAS patients might not benefit from aortic valve replacement (AVR) [2]. LGAS is usually found in patients with reduced LV ejection fraction (LVEF < 50%), but might also be present in patients with preserved LVEF ≥ 50% (so called paradoxical LGAS) [3]. Low-dose dobutamine stress echocardiography (DSE) is recommended as a tool to define the severity of aortic stenosis in patients with LGAS and reduced LVEF [4, 5]. To date, data regarding the utility of DSE for defining severity of LGAS in patients with preserved LVEF presenting with either paradoxical low flow or normal flow remain scanty. In the present study, we observed if DSE could also be useful to define AS severity in LGAS patients with preserved LVEF. We further explored the conventional echocardiographic markers suggestive of TS-LGAS in LGAS patients with reduced or preserved LVEF.

## Methods

### Study population

**Fig. 1:**
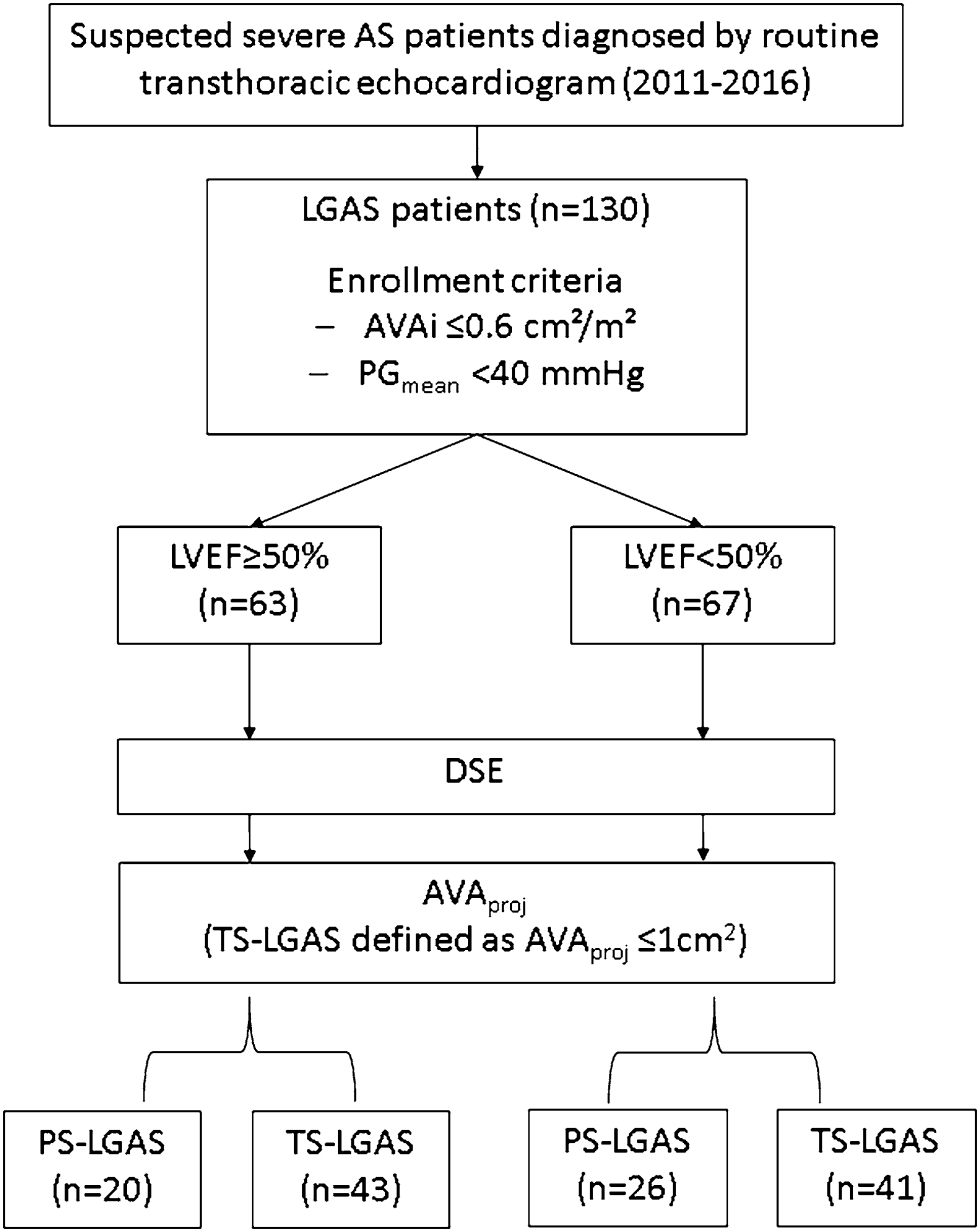
A flow-chart of the study protocol. *LGAS* low gradient aortic stenosis, *AVAi* indexed aortic valve area, *PG*_*mean*_ mean trans-aortic pressure gradient, *LVEF* left ventricular ejection fraction, *TS* true-severe, *PS* pseudo-severe

A total of 130 consecutive symptomatic low-gradient AS patients (aged 78 ± 8 years, 63.8% male), referred to the University Hospital Würzburg between January 2011 and December 2016, were included in this study. All patients underwent both standard transthoracic echocardiogram and DSE. Enrollment criteria included indexed aortic valve area (AVAi) ≤ 0.6 cm^2^/m^2^ and mean trans-aortic pressure gradient (PG_mean_) < 40 mmHg as assessed by transthoracic echocardiogram. The study protocol is shown in Fig. 1. The study was conducted in accordance to the Declaration of Helsinki and was approved by the Local Ethics Committee at the University of Würzburg (AZ 11/03 and 60/14). Informed consent was obtained from all patients or their guardians.

### Echocardiography

Echocardiographic examination was performed with GE Vingmed Vivid 7 or E9, Horten, Norway. Standard measurements on cardiac structural and functional parameters were made according to the current ASE guideline [6]. LV mass indexed to body surface area (LVMi) was calculated by the suggested formula [7]. End-diastolic and end-systolic volumes and LVEF were measured with the biplane Simpson method in the apical 4- and 2-chamber views. Septal and lateral mitral annular plane systolic excursion (MAPSE) and tricuspid annular plane systolic excursion (TAPSE) were measured by M-mode in the apical 4-chamber view. Systolic pulmonary artery pressure (SPAP) was derived from peak tricuspid regurgitation jet velocity using the simplified Bernoulli equation, in combination with the estimated right atrial pressure. LV diastolic function was assessed according to ASE guidelines for the assessment of diastolic function [8]. Tissue Doppler imaging was performed at the septal and lateral mitral annular sites enabling on-line derivation of myocardial systolic velocity (TDI-s′) and diastolic early velocity (e′) [9]. Speckle tracking imaging analysis was performed using EchoPAC software (GE, Horten, Norway) as described previously [10]. Longitudinal peak systolic strain (LS) of each segment was measured and global LS values were acquired by averaging strain rate and strain values of all 18 segments.

### Evaluation of AS severity by standard echocardiography

The diagnosis and classification of AS was made according to recent EAE/ASE recommendations [11]. Left ventricular outflow tract (LVOT) diameter was measured in the parasternal long-axis view focusing on the LVOT at baseline, and this value was also used to calculate the aortic valve area during DSE. The outer edge of the velocity spectrum obtained by continuous wave (CW) Doppler across the aortic valve (AV) was traced to obtain the maximum velocity (V_max_), maximum and mean trans-aortic pressure gradient (PG_mean_ and PG_max_), and AV velocity time integral (VTI). The subvalvular V_max_ (LVOT V_max_), VTI and stroke volume (SV) were obtained by tracing the outer edge of the velocity spectrum at the LVOT by the pulsed wave (PW) Doppler. Aortic valve area by continuity equation VTI [AVA (VTI)] was automatically calculated using the formula:

$$3.14/4*\{{\text{LVOT Dia}}{{\text{m}}\}^2}*\{{\text{LVOT~VTI}}\}/\{{\text{AV~VTI}}\}.$$

Valvuloarterial impedance (Zva) was calculated using the formula:

$${\text{Zva}}\left( {{\text{mmHg}}/{\text{ml}}/{{\text{m}}^{\text{2}}}} \right)={{\left\{ {{\text{systolic blood pressure}}+{\text{P}}{{\text{G}}_{{\text{mean}}}}} \right\}} \mathord{\left/ {\vphantom {{\left\{ {{\text{systolic blood pressure}}+{\text{P}}{{\text{G}}_{{\text{mean}}}}} \right\}} {\left\{ {{\text{body surface area indexed SV}}} \right\}}}} \right. \kern-0pt} {\left\{ {{\text{body surface area indexed SV}}} \right\}}}.$$

### Low-dose dobutamine stress echocardiography

Dobutamine infusion was started at a dose of 5 µg/kg/min, then increasing to 10, 15, and 20 µg/kg/min at 3-min intervals. Blood pressure and heart rate were recorded at rest and at the end of each stage of dobutamine dosage. Contractile reserve was defined as an increase in stroke volume (SV) of 20% or more [12]. The projected aortic valve area at normal transvalvular flow rate (AVA_proj_) was calculated using a simplified method with the following formula [13]: Simplified AVA_proj_ = AVA_rest_ + VC_simpl_ × (250-Q_rest_), where VC_simpl_ is the valve compliance and Q was computed by dividing SV by LV ejection time. VC_simpl_ was computed with the formula:

$${{\left( {{\text{AV}}{{\text{A}}_{{\text{peak}}}} - {\text{AV}}{{\text{A}}_{{\text{rest}}}}} \right)} \mathord{\left/ {\vphantom {{\left( {{\text{AV}}{{\text{A}}_{{\text{peak}}}} - {\text{AV}}{{\text{A}}_{{\text{rest}}}}} \right)} {\left( {{{\text{Q}}_{{\text{peak}}}} - {{\text{Q}}_{{\text{rest}}}}} \right)}}} \right. \kern-0pt} {\left( {{{\text{Q}}_{{\text{peak}}}} - {{\text{Q}}_{{\text{rest}}}}} \right)}},$$

where AVA_rest_ and AVA_peak_ are AVA at rest and peak DSE. Q_peak_ and Q_rest_ are Q at rest and peak DSE. TS-LGAS was defined as AVA_proj_ ≤1 cm^2^.

### Follow-up

Patients were followed up for a mean of 18 (12–27) months by clinical visit or telephone interview.

### Data analysis

Continuous variables are presented as mean ± standard deviation or median (interquartile range, IQR), as appropriate. Normal distribution of all continuous variables was tested by inspecting skewness, kurtosis, and Q–Q plots. Clinical and echocardiographic continuous variables between preserved and reduced LVEF groups and between PS-LGAS and TS-LGAS groups were compared using independent Student´s t test or Mann–Whitney U test as indicated. Categorical variables were expressed as percentages and were compared using a Chi square test or Fisher’s exact test, as appropriate. Multivariate logistic regression analysis was conducted to identify markers for differentiating TS-LGAS from PS-LGAS. Odds ratios (OR) with 95% confidence interval (CI) were assessed for indicating diagnostic performance. A two-tailed probability value < 0.05 was considered significant. Statistical analysis was performed using IBM SPSS, version 23 for Windows (IBM Corp., New York, USA).

## Results

### Clinical characteristics

Patients were divided into two subgroups: LVEF ≥ 50% group (n = 63) and LVEF < 50% group (n = 67). As shown in Table 1, the proportion of male patients was significantly higher in the LVEF < 50% group than in the LVEF ≥ 50% group (82.1% vs. 44.4%, P < 0.001). Prevalence of NYHA class III-IV was documented in 79.1% of LVEF < 50% patients and 55.5% of LVEF ≥ 50% patients (P = 0.003). The prevalence of diabetes (52.2% vs. 33.3%, P = 0.030) and coronary heart disease (71.6% vs. 49.2%, P = 0.009) was also significantly higher in the LVEF < 50% group than in the LVEF ≥ 50% group.

**Table 1:** Baseline clinical characteristics

	Total	LVEF ≥ 50%	LVEF < 50%	P value
	n = 130	n = 63	n = 67	
Age (years)	78 ± 8	79 ± 6	77 ± 8	0.117
Male [n (%)]	83 (63.8)	28 (44.4)	55 (82.1)	< 0.001
BMI (kg/m^2^)	27 ± 4	27 ± 4	27 ± 4	0.807
NYHA class [n (%)]				0.003
I	10 (7.7)	10 (15.9)	0 (0)	
II	32 (24.6)	18 (28.6)	14 (20.9)	
III	75 (57.7)	30 (47.6)	45 (67.2)	
IV	13 (10.0)	5 (7.9)	8 (11.9)	
Comorbidities [n (%)]				
Atrial fibrillation	59 (45.4)	28 (44.4)	31 (46.3)	0.835
Systemic hypertension	114 (87.7)	55 (87.3)	59 (88.1)	0.895
Diabetes mellitus	56 (43.1)	21 (33.3)	35 (52.2)	0.030
Obesity	28 (21.5)	16 (25.4)	12 (17.9)	0.299
Current smoking	24 (18.5)	9 (14.3)	15 (22.4)	0.234
Dyslipidemia	68 (52.3)	29 (46.0)	39 (58.2)	0.165
Coronary heart disease	79 (60.8)	31 (49.2)	48 (71.6)	0.009
CKD stage III-V	80 (61.5)	38 (60.3)	42 (62.7)	0.781
Respiratory disease	35 (26.9)	13 (20.6)	22 (32.8)	0.117
Malignancy	18 (13.8)	9 (14.3)	9 (13.4)	0.888
Stroke/TIA	16 (12.3)	9 (14.3)	7 (10.4)	0.506
Creatinine (mg/dl)	1.15 (0.99–1.70)	1.10 (0.93–1.53)	1.27 (1.00-1.81)	0.040
eGFR (ml/min/1.73qm)	57 (39–74)	57 (41–74)	57 (36–72)	0.633
C-reactive protein (mg/dl)	0.48 (0.18–1.04)	0.47 (0.14–0.97)	0.53 (0.29–1.29)	0.130
Clinical outcomes				
Follow-up duration (months)	18 (12 to 27)	20 (12 to 30)	16 (12 to 27)	0.226
All-cause death [n (%)]	28 (21.5)	10 (15.9)	18 (26.9)	0.128
AVR/TAVI [n (%)]	37 (28.5)/44 (33.8)	19 (30.2)/22 (34.9)	18 (26.9)/22 (32.8)	0.813
OP within 30-days [n (%)]	35 (43.2)	18 (46.3)	16 (40.0)	0.565

*BMI* body mass index, *NYHA* New York Heart Association, *CKD* chronic kidney disease, *eGFR* estimated glomerular filtration rate, *AVR* aortic valve replacement, *TAVI*, transcatheter aortic valve implantation

### Baseline echocardiographic characteristics

Mean LVEF was 60 ± 6% in the LVEF ≥ 50% group and 46 ± 4% in the LVEF < 50% group (Table 2). Patients with LVEF < 50% had larger LV, RV and LA cavities, thicker LV walls, lower septal and lateral MAPSE and TAPSE as compared to patients with LVEF ≥ 50% (all P < 0.05). AV Vmax, PG_mean_, LVOT V_max_, AV velocity ratio, and SV were significantly lower, while Zva was significantly higher in the LVEF < 50% group than in the LVEF ≥ 50% group (all P < 0.05). AVA VTI was similar between the LVEF LVEF ≥ 50% and < 50% groups (0.86 ± 0.14 vs. 0.84 ± 0.16cm^2^, P = 0.538). LV longitudinal systolic function parameters including septal and lateral TDI-s´as well as global and regional longitudinal strain (LS) at the basal septum were significantly lower in the LVEF < 50% group than that in the LVEF ≥ 50% group.

**Table 2:** Baseline echocardiographic characteristics

	Total	LVEF ≥ 50%	LVEF < 50%	P value
	n = 130	n = 63	n = 67	
LVEDD (mm)	50 ± 7	45 ± 5	55 ± 6	< 0.001
IVSd (mm)	11.2 ± 1.3	10.7 ± 1.1	11.7 ± 1.3	< 0.001
LVPWd (mm)	10.8 ± 1.2	10.4 ± 1.0	11.2 ± 1.2	< 0.001
LAD (mm)	44 ± 6	43 ± 7	46 ± 42	0.002
LVEF (%)	47 ± 15	60 ± 6	46 ± 4	< 0.001
Septal MAPSE (mm)	6.8 ± 2.5	8.2 ± 2.2	5.4 ± 1.9	< 0.001
lateral MAPSE (mm)	8.4 ± 3.1	10.2 ± 2.7	6.8 ± 2.4	< 0.001
TAPSE (mm)	16.5 ± 4.9	18.5 ± 4.6	14.6 ± 4.4	< 0.001
RVD_basal (mm)	35 ± 7	34 ± 7	37 ± 7	0.011
RVD_mid (mm)	31 ± 8	30 ± 8	32 ± 7	0.165
RAA (cm^2^)	20 ± 7	19 ± 7	21 ± 7	0.284
E (cm/s)	101 ± 37	105 ± 41	97 ± 31	0.238
DT (ms)	194 ± 83	212 ± 88	176 ± 74	0.013
E′ (cm/s)	5.0 ± 2.2	5.2 ± 2.1	4.9 ± 2.2	0.374
E/E′	21 ± 9	21 ± 10	22 ± 8	0.354
Diastolic function [n (%)]				0.127
Normal	1 (0.8)	1 (1.6)	0	
Grade I	34 (26.2)	21 (33.3)	13 (19.4)	
Grade II	59 (45.4)	28 (44.4)	31 (46.3)	
Grade III	36 (27.7)	13 (20.6)	23 (34.3)	
Moderate or severe MR [n (%)]	33 (25.4)	13 (20.6)	20 (29.9)	0.228
Moderate or severe AR [n (%)]	23 (17.7)	11 (17.5)	12 (17.9)	0.946
Moderate or severe TR [n (%)]	37 (28.5)	17 (27.0)	20 (29.9)	0.717
SPAP (mmHg)	43 ± 17	43 ± 18	43 ± 16	0.944
LVOT (mm)	22.4 ± 1.6	21.7 ± 1.4	23.1 ± 1.4	< 0.001
AV V_max_ (m/s)	3.4 ± 0.4	3.5 ± 0.3	3.3 ± 0.4	0.004
AV PG_mean_ (mmHg)	29.4 ± 6.1	31.0 ± 5.7	27.9 ± 6.2	0.003
LVOT V_max_ (m/s)	0.74 ± 0.14	0.82 ± 0.12	0.67 ± 0.12	< 0.001
AV velocity ratio	0.22 ± 0.04	0.24 ± 0.04	0.20 ± 0.04	< 0.001
AVA_VTI (cm^2^)	0.85 ± 0.15	0.86 ± 0.14	0.84 ± 0.16	0.538
Indexed AVA_VTI (cm^2^/m^2^)	0.45 ± 0.08	0.47 ± 0.07	0.44 ± 0.08	0.012
SVi (ml/m^2^)	34.6 ± 9.2	40.0 ± 9.2	29.5 ± 5.6	< 0.001
Zva (mmHg/ml/m^2^)	4.7 ± 1.4	4.2 ± 1.0	5.2 ± 1.5	< 0.001
Septal TDI-s´ (cm/s)	3.8 ± 1.3	4.4 ± 1.2	3.2 ± 1.0	< 0.001
lateral TDI-s´ (cm/s)	4.6 ± 1.5	5.2 ± 1.4	4.1 ± 1.5	< 0.001
Longitudinal strain (%)				
GLS_4ch	11.4 ± 4.8	14.9 ± 3.9	8.2 ± 2.9	< 0.001
GLS_2ch	11.6 ± 4.7	14.7 ± 3.9	8.6 ± 3.2	< 0.001
GLS_3ch	11.5 ± 4.8	14.7 ± 4.0	8.7 ± 3.4	< 0.001
GLS_average	11.5 ± 4.5	14.7 ± 3.6	8.5 ± 2.8	< 0.001
Basal septal LS	8.3 ± 4.0	10.1 ± 3.6	6.6 ± 3.6	< 0.001

*LVEDD* left ventricular end-diastolic dimension, *IVSd* end-diastolic interventricular septal thickness, *LVPWd* end-diastolic left ventricular posterior wall thickness, *LVEF* left ventricular ejection fraction, *MAPSE* mitral annular plane systolic excursion, *TAPSE* tricuspid annular plane systolic excursion, *RVD* right ventricular dimension, *RAA* end-systolic right atrial area, *E* mitral inflow early filling velocity, *DT* deceleration time of early filling, *E*′ early diastolic mitral annular velocity, *E*/*E*′ the ratio between mitral inflow early filling velocity and mitral annular velocity, *MR* mitral regurgitation, *AR* aortic regurgitation, *TR* tricuspid regurgitation, *SPAP* systolic pulmonary artery pressure, *LVOT* left ventricular outflow tract, *AVA_VTI* aortic valve area calculated by the velocity time integral, *AV* aortic valve, *Vmax* maximum velocity by continuous-wave Doppler, *PG*_*mean*_ mean transvalvular pressure gradient, *SVi* stroke volume indexed to body surface area, *Zva*, valvuloarterial impedance; *TDI-s´* tissue-Doppler imaging derived mitral annular systolic peak velocity, *GLS* global longitudinal strain

### DSE

DSE results in the patients with LVEF ≥ 50% and LVEF < 50% are shown in Tables 3 and 4. Forty-three out of 63 (68.3%) in the LVEF ≥ 50% group and 41 out of 67 (61.2%) patients in the LVEF < 50% group were diagnosed as TS-LGAS by DSE. AV Vmax, PG_mean_, LVOT V_max_, AV velocity ratio, and AVA VTI significantly increased during DSE both in LVEF ≥ 50% and < 50% groups (all P < 0.05, Tables 3, 4). Systolic blood pressure remained unchanged while peak diastolic blood pressure decreased in both groups during DSE.

**Table 3:** Low-dose dobutamine stress echocardiographic characteristics

	Total	LVEF ≥ 50%	LVEF < 50%	P value
	n = 130	n = 63	n = 67	
True-severe AS [n (%)]	84 (64.6)	43 (68.3)	41 (61.2)	0.400
Pseudo-severe AS [n (%)]	46 (35.4)	20 (31.7)	26 (38.8)	
LV flow reserve (ΔSV ≥ 20%) [n (%)]	76 (58.5)	32 (50.8)	44 (65.7)	0.085
Rest SBP (mmHg)	126 ± 21	131 ± 21	120 ± 20	0.002
Peak SBP (mmHg)	125 ± 28	132 ± 29	118 ± 25	0.007
Δ SBP (%)	0 (– 14 to 12)	– 1 (– 15 to 13)	0 (– 13 to 8)	0.658
Rest DBP (mmHg)	66 ± 13	66 ± 13	65 ± 19	0.820
Peak DBP (mmHg)	58 ± 14*	58 ± 15*	58 ± 13*	0.739
Δ DBP (%)	– 11 (-23 to 0)	– 8 (– 21 to 0)	– 13 (– 24 to 0)	0.955
Rest HR (beats/min)	70 ± 12	67 ± 11	72 ± 12	0.006
Peak HR (beats/min)	92 ± 20*	94 ± 19*	91 ± 20*	0.385
Δ HR (%)	29 (13 to 50)	40 (18 to 61)	20 (9 to 44)	0.001
Rest LVEF (%)	47 ± 16	61 ± 8	34 ± 8	< 0.001
Peak LVEF (%)	58 ± 17*	71 ± 8*	45 ± 12*	< 0.001
Δ LVEF (%)	24 (12 to 36)	16 (8 to 25)	34 (20 to 51)	< 0.001
Rest LVSV (ml)	68 ± 17	74 ± 16	62 ± 16	< 0.001
Peak LVSV (ml)	79 ± 20*	85 ± 20*	73 ± 19*	< 0.001
Δ LVSV (%)	23 (3 to 41)	20 (– 2 to 38)	27 (4 to 42)	0.069
Rest AV V_max_ (m/s)	3.4 ± 0.4	3.5 ± 0.4	3.3 ± 0.3	0.001
Peak AV V_max_ (m/s)	4.2 ± 0.6*	4.4 ± 0.5*	4.0 ± 0.5*	< 0.001
Δ AV V_max_ (%)	23 (11 to 32)	27 (15 to 34)	22 (8 to 32)	0.148
Rest PG_mean_ (mmHg)	30 ± 6	32 ± 6	29 ± 6	0.003
Peak PG_mean_ (mmHg)	45 ± 12*	48 ± 11*	42 ± 12*	0.005
Δ PG_mean_ (%)	45 (22 to 67)	48 (29 to 75)	44 (19 to 66)	0.685
Rest LVOT V_max_ (m/s)	0.7 ± 0.2	0.8 ± 0.1	0.7 ± 0.1	< 0.001
Peak LVOT V_max_ (m/s)	1.0 ± 0.3*	1.1 ± 0.2*	0.9 ± 0.2*	< 0.001
Δ LVOT V_max_ (%)	34 (9 to 49)	36 (14 to 52)	25 (4 to 43)	0.063
Rest AV velocity ratio	0.22 ± 0.04	0.24 ± 0.04	0.20 ± 0.04	< 0.001
Peak AV velocity ratio	0.24 ± 0.06*	0.25 ± 0.06*	0.22 ± 0.04*	0.001
Δ AV velocity ratio (%)	5 (– 9 to 19)	11 (– 6 to 18)	2 (– 11 to 21)	0.479
Rest AVA_VTI (cm^2^)	0.88 ± 0.18	0.89 ± 0.15	0.86 ± 0.21	0.301
Peak AVA_VTI (cm^2^)	0.98 ± 0.27*	1.04 ± 0.24*	0.92 ± 0.27*	0.009
Δ AVA_VTI (%)	11 (– 2 to 28)	15 (3 to 29)	4 (– 9 to 25)	0.047
Rest flow rate	0.22 ± 0.05	0.24 ± 0.04	0.21 ± 0.05	0.003
Peak flow rate	0.33 ± 0.10	0.36 ± 0.10	0.30 ± 0.08	< 0.001
Δ flow rate (%)	46 (28 to 62)	49 (33 to 71)	45 (20 to 60)	0.151
AVA_proj_ (cm^2^)	0.90 ± 0.19	0.89 ± 0.14	0.91 ± 0.25	0.588

*SBP* systolic blood pressure, *DBP* diastolic blood pressure, *HR* heart rate, *LVEF* left ventricular ejection fraction, *PG*_*mean*_ mean transvalvular pressure gradient, *SVi* stroke volume indexed to body surface area, *AVA_VTI* aortic valve area calculated by the velocity time integral, *LGSAS* low-gradient severe aortic stenosis, *LGMAS* low-gradient moderate aortic stenosis, *AVA*_*proj*_: projected aortic valve area at normal transvalvular flow rate

*P < 0.05 vs. respective parameters at rest

**Table 4:** DSE characteristics in patients with PS- and TS-LGAS patients

	LVEF ≥ 50%		LVEF < 50%	
	PS-LGAS (n = 20)	TS-LGAS (n = 43)	PS-LGAS n = 26	TS-LGAS n = 41
Rest AV V_max_ (m/s)	3.5 ± 0.4	3.6 ± 0.4	3.3 ± 0.4	3.3 ± 0.3
Peak AV V_max_ (m/s)	4.2 ± 0.5	4.5 ± 0.5*	3.7 ± 0.4	4.2 ± 0.5*
Δ AV V_max_ (%)	18 (13 to 31)	29 (17 to 36)*	13 (2 to 24)	26 (11 to 33)*
Rest PG_mean_ (mmHg)	31 ± 7	32 ± 6	28 ± 6	29 ± 6
Peak PG_mean_ (mmHg)	43 ± 11	50 ± 11*	35 ± 8	46 ± 12*
Δ PG_mean_ (%)	39 (16 to 63)	54 (36 to 77)	30 (4 to 49)	50 (28 to 84)*
Rest LVOT V_max_ (m/s)	0.9 ± 0.1	0.8 ± 0.1	0.7 ± 0.1	0.6 ± 0.1*
Peak LVOT V_max_ (m/s)	1.2 ± 0.2	1.1 ± 0.2*	1.0 ± 0.2	0.8 ± 0.2*
Δ LVOT V_max_ (%)	38 (22 to 60)	34 (11 to 50)	36 (10 to 51)	23 (3 to 41)
Rest AV velocity ratio	0.25 ± 0.04	0.23 ± 0.04	0.22 ± 0.03	0.19 ± 0.04*
Peak AV velocity ratio	0.29 ± 0.05	0.24 ± 0.05*	0.26 ± 0.07	0.19 ± 0.04*
Δ AV velocity ratio (%)	17 (5 to 26)	4 (– 12 to 16)*	14 (– 2 to 36)	– 4 (– 16 to 11)*
Rest AVA_VTI (cm^2^)	0.97 ± 0.14	0.86 ± 0.14*	0.94 ± 0.22	0.81 ± 0.18*
Peak AVA_VTI (cm^2^)	1.23 ± 0.24	0.96 ± 0.20*	1.12 ± 0.28	0.80 ± 0.19*
Δ AVA_VTI (%)	20 (10 to 45)	14 (– 1 to 25)*	24 (– 1 to 39)	0 (– 11 to 11)*
Rest septal MAPSE (mm)	9.5 ± 2.2	7.9 ± 2.2*	5.2 ± 1.6	5.2 ± 1.6
Peak septal MAPSE (mm)	10.2 ± 2.7	9.2 ± 2.6	6.2 ± 2.3	6.2 ± 2.2
Δ septal MAPSE (%)	4 (– 7 to 18)	17 (0 to 33)	17 (0 to 37)	25 (0 to 50)
Rest lateral MAPSE (mm)	11.6 ± 2.2	9.8 ± 2.6*	7.1 ± 2.3	6.4 ± 2.4
Peak lateral MAPSE (mm)	12.8 ± 3.4	11.2 ± 2.7	9.5 ± 3.2	8.6 ± 3.3
Δ lateral MAPSE (%)	12 (0 to 27)	12 (0 to 28)	33 (9 to 61)	37 (0 to 75)
Rest TAPSE (mm)	18.2 ± 3.9	17.9 ± 5.0	14.9 ± 4.9	14.0 ± 5.4
Peak TAPSE (mm)	18.8 ± 5.6	18.5 ± 5.3	16.4 ± 6.0	14.7 ± 5.5
Δ TAPSE (%)	5 (– 19 to 23)	0 (– 8 to 17)	12 (– 8 to 20)	8 (– 8 to 18)
Rest SPAP (mmHg)	42 ± 16	41 ± 14	37 ± 14	46 ± 15*
Peak SPAP (mmHg)	55 ± 16	54 ± 15	43 ± 17	57 ± 15*
Δ SPAP (%)	30 (11 to 51)	34 (12 to 54)	15 (0 to 29)	18 (10 to 40)
AVA_proj_ (cm^2^)	1.06 ± 0.04	0.84 ± 0.11*	1.21 ± 0.22	0.83 ± 0.19*

Abbreviations as shown in Table 3

*P < 0.05 vs. PS-LGAS

In the LVEF ≥ 50% group, baseline AVA_VTI (0.86 ± 0.14 vs. 0.97 ± 0.14 cm^2^, P = 0.007) and baseline MAPSE (septal: 7.9 ± 2.2 vs. 9.5 ± 2.2 mm, P = 0.009; lateral: 9.8 ± 2.6 vs. 11.6 ± 2.2 mm, P = 0.011) were significantly lower in the TS-LGAS subgroup than in the PS-LGAS subgroup. During DSE, AV velocity ratio was significantly increased in the PS-LGAS group (baseline 0.25 ± 0.04 vs. peak 0.29 ± 0.05, P < 0.001), while remained unchanged in the TS-LGAS group (baseline 0.23 ± 0.04 vs. peak 0.24 ± 0.05, P = 0.531). AVA_VTI was significantly increased during DSE in the PS-LGAS group (baseline 0.97 ± 0.14 vs. peak 1.23 ± 0.24 cm^2^, P < 0.001) and in the TS-LGAS group (baseline 0.86 ± 0.14 vs. peak 0.96 ± 0.20 cm^2^, P = 0.001).

In the LVEF < 50% group, baseline AV velocity ratio (0.19 ± 0.04 vs. 0.22 ± 0.03, P = 0.006) and baseline AVA_VTI (0.81 ± 0.18 vs. 0.94 ± 0.22 cm^2^, P = 0.008) were significantly lower, while baseline SPAP (46 ± 15 vs. 37 ± 14 mmHg, P = 0.031) was significantly higher in the TS-LGAS group than in the PS-LGAS group. AV velocity ratio (baseline 0.22 ± 0.03 vs. peak 0.26 ± 0.07, P = 0.001) and AVA_VTI (baseline 0.94 ± 0.22 vs. peak 1.12 ± 0.28 cm^2^, P = 0.005) were significantly increased in the PS-LGAS group, while remained unchanged in the TS-LGAS group during DSE (AV velocity ratio: baseline 0.19 ± 0.04 vs. peak 0.19 ± 0.05, P = 0.352; AVA_VTI: baseline 0.81 ± 0.18 vs. peak 0.80 ± 0.19 cm^2^, P = 0.742).

All patients with baseline AVAi ≤ 0.3cm^2^/m^2^ were identified as TS-LGAS by DSE (n = 5). In the LVEF ≥ 50% group, 100%, 65.6% and 57.1% of patients with a baseline AVAi of 0.31–0.4, 0.41–0.5 and 0.51–0.6cm^2^/m^2^ were identified as TS-LGAS, respectively. In the LVEF < 50% group, 78.9%, 65.5%, and 25.0% of patients with a baseline AVAi of 0.31–0.4, 0.41–0.5, and 0.51–0.6cm^2^/m^2^ were identified as TS-LGAS, respectively. Baseline AVAi ≤ 0.4cm^2^/m^2^ was highly suggestive of TS-LGAS in LGAS patients with LVEF ≥ 50% (specificity 100% and sensitivity 23%).

**Fig. 2:**
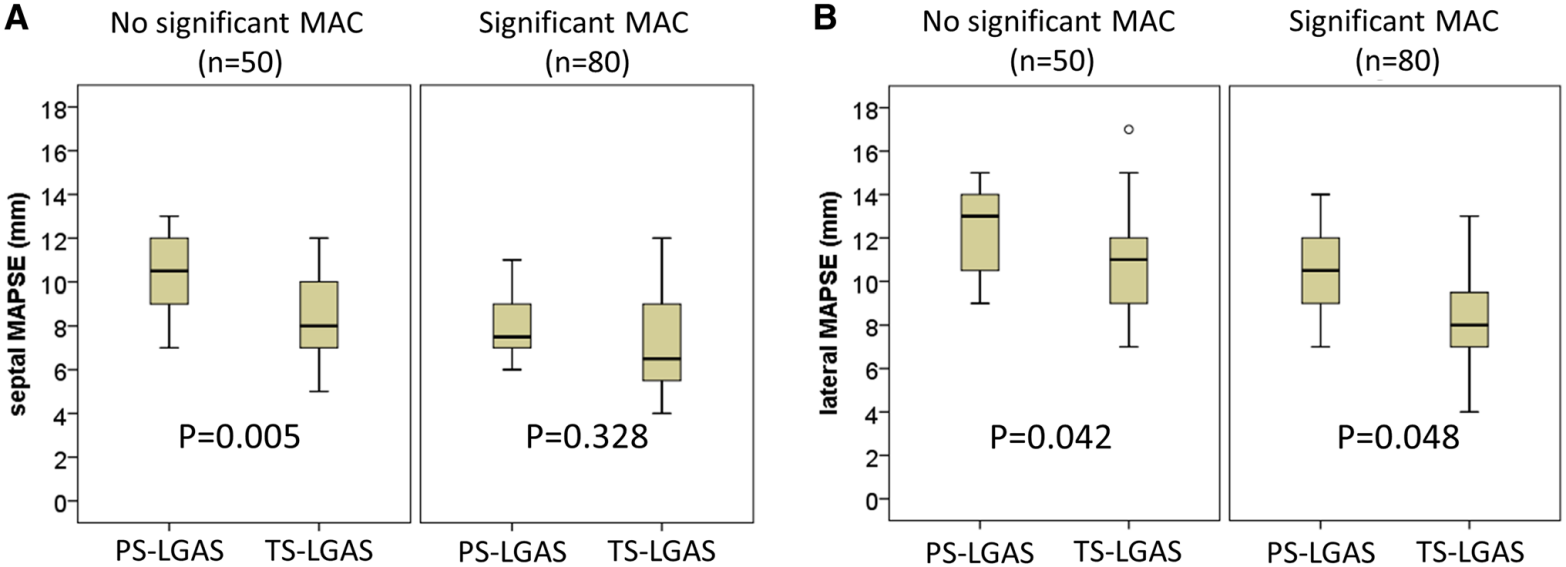
Septal (**a**) and lateral (**b**) mitral annular plane systolic excursion (MAPSE) in low-gradient aortic stenosis (LGAS) patients with and without significant mitral annular calcification (MAC)

Significant mitral annular calcification (MAC) was found in 80 out of 130 (61.5%) patients. As shown in Fig. 2a, baseline septal MAPSE was significantly lower in the TS-LGAS group as compared to the PS-LGAS group in LVEF ≥ 50% patients without significant MAC (8.3 ± 2.1 vs. 10.5 ± 2.0 mm, P = 0.005). Lateral MAPSE was significantly lower in TS-LGAS group as compared to PS-LGAS group in LVEF ≥ 50% patients without (10.7 ± 2.4 vs. 12.3 ± 1.9 mm, P = 0.042) and with significant MAC (8.4 ± 2.2 vs. 10.5 ± 2.3 mm, P = 0.048; Fig. 2b).

### Echocardiographic markers suggestive of TS-LGAS

Parameters, which are significantly associated with TS-LGAS with a statistic difference (p < 0.05) between the PS-LGAS and TS-LGAS groups either in the subgroup of LVEF ≥ 50% or in the subgroup of LVEF < 50% (Tables 4, 5), were tested in the multivariable binary regression models. Multivariable logical regression models (Table 6) showed that baseline AVA_VTI was an independent determinant of TS-LGAS both in the LVEF ≥ 50% and < 50% groups after adjusted for age and sex (LVEF ≥ 50%: OR 0.45, P = 0.004; LVEF < 50%: OR 0.55, P = 0.005). Besides, lower septal and lateral MAPSE (adjusted OR 0.72–0.75, P = 0.013–0.016) as well as septal TDI-s´ (adjusted OR 0.53, P = 0.010) were significantly associated with TS-LGAS in patients with LVEF ≥ 50%. Higher SPAP and lower AV velocity ratio were associated with TS-LGAS in patients with LVEF < 50% (SPAP: OR 1.43, P = 0.045; AV velocity ratio: OR 0.21, P = 0.035).

**Table 5:** Baseline left ventricular longitudinal function characteristics in patients with PS- and TS-LGAS patients

	LVEF ≥ 50%		LVEF < 50%	
	PS-LGAS (n = 20)	TS-LGAS (n = 43)	PS-LGAS n = 26	TS-LGAS n = 41
Septal MAPSE (mm)	9.5 ± 2.2	7.9 ± 2.2*	5.2 ± 1.6	5.2 ± 1.6
Lateral MAPSE (mm)	11.6 ± 2.2	9.8 ± 2.6*	7.1 ± 2.3	6.4 ± 2.4
Septal TDI-s´ (cm/s)	5.0 ± 1.3	4.1 ± 1.1*	3.3 ± 1.1	3.0 ± 0.9
Lateral TDI-s´ (cm/s)	5.5 ± 1.4	5.2 ± 1.4	4.1 ± 1.4	4.1 ± 1.6
GLS (%)	14.4 ± 3.6	14.9 ± 3.7	9.0 ± 2.5	8.3 ± 3.0

Abbreviations as shown in Table 2

*P < 0.05 vs. PS-LGAS

**Table 6:** Odds ratio of echocardiographic determinants for low-gradient severe aortic stenosis

	LVEF ≥ 50% (n = 63)			LVEF < 50% (n = 67)		
	Age and sex adjusted OR	95% CI	P value	Age and sex adjusted OR	95% CI	P value
Septal MAPSE (per 1 mm increase)	0.72	0.56–0.93	0.013	0.98	0.72–1.34	0.906
Lateral MAPSE (per 1 mm increase)	0.75	0.59–0.95	0.016	0.92	0.74–1.15	0.473
Septal TDI-s´ (per 1 cm/s increase)	0.53	0.33–0.86	0.010	0.73	0.44–1.21	0.219
SPAP (per 10 mmHg increase)	0.95	0.70–1.28	0.732	1.43	1.01–2.04	0.045
AVA_VTI (per 0.1cm^2^ increase)	0.45	0.26–0.78	0.004	0.55	0.36–0.83	0.005
AV velocity ratio (per 0.1 increase)	0.29	0.06–1.29	0.286	0.21	0.05–0.90	0.035

*CI* confidence interval, abbreviations as shown in Table 2

### Clinical Follow up results

During follow-up, 28 (21.5%) patients died, 10 (15.9%) in LVEF ≥ 50% group [3 with TS-LGAS (conservative therapy) and 2, 3, 7 with PS-LGAS (2 with conservative therapy, 3 surgical aortic valve replacement, and 2 transcatheter aortic valve implantation)], and 18 (26.9%) in LVEF < 50% group [10 with TS-LGAS (6 conservative therapy, 1, 3, 8 surgical aortic valve replacement, and 3 transcatheter aortic valve implantation) and 8 with PS-LGAS (6 with conservative therapy, 1 surgical aortic valve replacement, and 1 transcatheter aortic valve implantation)].

## Discussion

The major findings of the present study are: (1) DSE appears to also be a helpful tool of defining true-severe low gradient aortic stenosis in patients with LVEF ≥ 50%; (2) Baseline AVA derived from transthoracic echocardiography is an independent determinant of TS-LGAS in both patients with LVEF ≥ 50% as well as LVEF < 50%; (3) Reduced septal and lateral MAPSE as well as septal TDI-s´ are associated with TS-LGAS in patients with LVEF ≥ 50% while higher SPAP and lower AV velocity ratio are independently associated with TS-LGAS in patients with LVEF < 50%.

### Value of DSE in LGAS patients with reduced and preserved LVEF

Patients with low gradient severe aortic stenosis (LGAS) and preserved LVEF (AVA <1 cm^2^, PG_mean_ <40 mmHg, LVEF >50%) are increasingly recognised in clinical practice. DSE is recommended to define the severity of aortic stenosis in patients with LGAS and reduced LVEF [4, 5]. Our results showed that DES is also useful in defining TS- from PS-LGAS in patients with LVEF < 50% (Table 4). This finding is in line with a recent study, which demonstrated that DSE might serve as a valuable tool to distinguish TS-LGAS from PS-LGAS in patients with paradoxical LGAS [14].

There were 20 out of 63 (31.7%) LGAS patients with preserved LVEF presenting with paradoxical low-flow LGAS (SVi ≤ 35 ml/m^2^) in our cohort. We compared other related parameters as well as the response on DSE between the two subgroups (low-flow vs. normal-flow LGAS). The data show that LV cavity in these patients was smaller than in patients with normal-flow LGAS (left ventricular end-diastolic dimension 43 ± 4 vs. 46 ± 5 mm, P = 0.021). Moreover, DSE seems also to be feasible to identify the TS-AS patients in the low-flow LGAS subgroup as in the normal-flow LGAS subgroup (Supplementary Table 1). Future studies with larger patient cohort are warrant to verify these results.

### Echocardiographic determinant of TS-LGAS in patients with preserved LVEF

In line with previous findings from our group [15] and others [16], the present study results demonstrate that reduced MAPSE is suggestive of TS-LGAS in patients with LVEF > 50%. A previous study also showed that MAPSE correlated with aortic valve area (Spearman *r* = 0.18, P = 0.02) in 205 asymptomatic AS patients with LVEF ≥ 50% [17]. In another study, Rydberg and colleagues found that left atrioventricular plane displacement (MAPSE), but not left ventricular ejection fraction, was influenced by the degree of aortic stenosis [16]. In patients with LGAS and preserved LVEF, LVEF is maintained at normal range at the expense of high LV end-diastolic pressure and volume. In this circumference, LV cannot recruit additional preload because the maximum sarcomere length is already reached and the hypertrophied stiff ventricle could not tolerate additional filling load [18]. This might explain why reduced MAPSE, as a function of LV hemodynamic load, was revealed as the most sensitive marker of TS-LGAS in LGAS patients with LVEF > 50%. Additionally, reduction in TDI-s´, another parameter reflecting LV longitudinal dysfunction, is also suggestive of TS-LGAS in AS patients with LVEF ≥ 50%.

### Echocardiographic determinants of TS-LGAS in patients with reduced LVEF

In LGAS patients with reduced LVEF, the disease features include both AS and heart failure. Ventricular remodeling (myocyte hypertrophy and myocardial fibrosis), systolic and diastolic dysfunction, in the setting of valvular stenosis and heart failure scenarios, contributes to the progressively elevated LV filling pressure, and/or left atrial pressure, which in turn would transmit to the pulmonary vasculature, inducing pulmonary venous congestion and pulmonary hypertension, so called WHO Group II - pulmonary hypertension [19, 20]. A previous study showed that pulmonary hypertension was presented in the majority of patients with severe aortic stenosis, and ejection fraction serves as one of the correlates of pulmonary hypertension in patients with severe aortic stenosis [21]. Another study indicated that both smaller aortic valve area and lower LVEF were responsible for the presence of pulmonary hypertension in a cohort of 626 patients with severe aortic stenosis [22]. In line with above findings, we found that increased systolic pulmonary pressure is an independent determinant of TS in LGAS patients with reduced LVEF. Another finding of the present study is that lower AV velocity ratio is related to the presence of true severe in LGAS patients with reduced LVEF. This parameter is only recommended in the EAE/ASE recommendations, a value of < 0.25 is suggestive of severe AS [23]. In our cohort, this value ranged from 0.23 at rest and 0.24 during DSE in TS-LGAS patients with LVEF > 50%, and 0.19 at rest and 0.19 during DSE in TS-LGAS patients with reduced LVEF. A previous study reported that velocity ratios could not only define the severity of AS, but also predict outcomes in patients with TS-LGAS and preserved LVEF [24]. Our results suggest AV velocity ratio could be used as an independent predictor for defining TS in LGAS patients with reduced LVEF.

## Limitations

This retrospective study was performed monocentric and the number of patients included in the sub-cohorts was relatively small. The results of the present study might thus be affected by patient selection bias. Future studies with large patient cohort are warranted to validate the results obtained from this study. In this study, projected aortic valve area assessed by DSE was used to define the severity of AS [25]. Other imaging modalities may aid in the diagnosis of severe AS. Macroscopic evaluation of the valve at the time of valve replacement or measurement of aortic valve calcium score by multislice computed tomography could provide additional accuracy to differ TS- from PS-AS [26]. Due to the lack of available CT data for AV calcium score in our cohort, the severity of AS was only determined by projected aortic valve area. This study limitation should be considered in the interpretation of results derived from the current study.

### Clinical implications

Our study results suggest that DSE enables evaluation of the severity of AS both in LGAS patients with reduced LVEF and in those with preserved LVEF, which is consistent with what has been demonstrated by previous studies [13, 26]. In the present study, we further explored the echocardiographic determinants suggestive of TS-LGAS. Reduced MAPSE and TDI-s´ are suggestive of TS in LGAS patients with preserved LVEF, while increased SPAP and lower AV velocity ratio are suggestive of TS in LGAS patients with reduced LVEF. These findings provide incremental information on the diagnosis and therapy decision for LGAS patients, particularly in those patients who cannot tolerate DSE due to the presence of contraindications. Patients with related echocardiographic features should be carefully evaluated for the AS severity with alternative complementary imaging modalities, such as measurement of aortic valve calcium score assessed by multislice computed tomography.

## Conclusions

DSE is valuable for staging of LGAS in patients with both reduced and preserved LVEF. Low longitudinal LV function (MAPSE) and septal TDI-s´ are associated with TS-LGAS in patients with preserved LVEF, and high trans-tricuspid pressure gradient (SPAP) and low AV velocity ratio are associated with TS-LGAS in patients with reduced LVEF.

## Electronic supplementary material

Below is the link to the electronic supplementary material.

Supplementary material 1 (DOCX 14 KB)
