# Supplementary material for: Value of low-dose dobutamine stress echocardiography on defining true severe low gradient aortic stenosis in patients with preserved left ventricular ejection fraction
Source: Int J Cardiovasc Imaging. 2018 Jul 23;34(12):1877–87. doi: 10.1007/s10554-018-1416-z (PMC6245091; doi:10.1007/s10554-018-1416-z)
Supplement: Supplementary file 1 — Supplementary material 1 (DOCX 14 KB) [file 10554_2018_1416_MOESM1_ESM.docx]

**Supplementary table 1. DSE characteristics in LVEF≥50%and LGAS patients with normal and reduced stroke volume index (SVi)**

|  | SVi>35ml/m² | | SVi≤35ml/m² | |
| --- | --- | --- | --- | --- |
|  | PS-LGAS  n=14 | TS-LGAS  n=29 | PS-LGAS  n=6 | TS-LGAS  n=14 |
| Rest AV V_max_ (m/s) | 3.5±0.2 | 3.6±0.3 | 3.2±0.3 | 3.4±0.4 |
| Peak AV V_max_ (m/s) | 4.3±0.5 | 4.6±0.5 | 3.8±0.5 | 4.2±0.5 |
| Δ AV V_max_ (%) | 20 (13 to 30) | 29 (15 to 37) | 17 (0 to 33) | 28 (17 to 32) |
|  |  |  |  |  |
| Rest PG_mean_ (mmHg) | 32±5 | 34±4 | 26±6 | 29±8 |
| Peak PG_mean_ (mmHg) | 47±10 | 53±10 | 33±7 | 43±10* |
| Δ PG_mean_ (%) | 42 (19 to 69) | 59 (34 to 81) | 25 (5 to 59) | 53 (39 to 67) |
|  |  |  |  |  |
| Rest LVOT V_max_ (m/s) | 0.9±0.1 | 0.9±0.1 | 0.8±0.2 | 0.6±0.1 |
| Peak LVOT V_max_ (m/s) | 1.3±0.2 | 1.1±0.2 | 1.1±0.2 | 0.9±0.3 |
| Δ LVOT V_max_ (%) | 52 (35 to 65) | 33 (10 to 52)* | 27 (10 to 42) | 37 (13 to 45) |
|  |  |  |  |  |
| Rest AV velocity ratio | 0.24±0.04 | 0.24±0.04 | 0.26±0.05 | 0.22±0.05 |
| Peak AV velocity ratio | 0.29±0.05 | 0.24±0.05* | 0.29±0.05 | 0.22±0.05* |
| Δ AV velocity ratio (%) | 22 (11 to 29) | 3 (-13 to 17)* | 8 (2 to 17) | 7 (-13 to 17) |
|  |  |  |  |  |
| Rest AVA_VTI (cm²) | 0.95±0.13 | 0.90±0.12 | 1.01±0.16 | 0.78±0.15* |
| Peak AVA_VTI (cm²) | 1.23±0.25 | 1.03±0.17* | 1.23±0.23 | 0.81±0.17* |
| Δ AVA_VTI (%) | 20 (13 to 47) | 14 (5 to 28)* | 20 (0 to 48) | 10 (-6 to 22) |
|  |  |  |  |  |
| AVA_proj_ (cm²) | 0.96±0.09 | 0.91±0.12 | 1.05±0.03 | 0.85±0.25* |

* P<0.05 vs. PS-LGAS; abbreviations as shown in table 3.
